# Supplementary material for: A novel transposable element-based authentication protocol for Drosophila cell lines
Source: G3 (Bethesda). 2021 Nov 25;12(2):jkab403. doi: 10.1093/g3journal/jkab403 (PMC9210319; doi:10.1093/g3journal/jkab403)
Supplement: jkab403_Supplementary_Data [file jkab403_supplementary_data.zip › GENETICS-G3-2021-402803-s05.docx]

| **Primer Name** | **TE-specificity** | **Sequence** |
| --- | --- | --- |
| TE297 Reaction A Rev205 | 297 | TGGGTTCTTAAGATTAGGTAGCGTTGAAAG |
| TE297 Reaction A Nest PCR Rev121 | 297 | **GTTCAGACGTGTGCTCTTCCGATCT**GGNCTTTAGGATGTTTACAAAGAACGCTGC |
| TE297 Reaction B For6819 | 297 | CGAGGCTCTCCCGAAATACAAATATTGTTC |
| TE297 Reaction B Nest PCR For6896 | 297 | **GTTCAGACGTGTGCTCTTCCGATCT**TTAAGCTGAGATCCAAAGAATAAAGWCGTG |
| TE1731 Reaction A Rev73 | 1731 | TACAGTTCATGTGTATGTATGTATGTTCTC |
| TE1731 Reaction A Nest PCR Rev13 | 1731 | **GTTCAGACGTGTGCTCTTCCGATCT**CCTATATTCAACACACATGTGGGCATTG |
| TE1731 Reaction B For4446 | 1731 | GCATGAAGCTGGCATTTTTATGTGTATCAG |
| TE1731 Reaction B Nest PCR For4513 | 1731 | **GTTCAGACGTGTGCTCTTCCGATCT**TCGGCTCAAGACTTTTTATTTCGCGTTTAC |
| TEmdg1 Reaction A Rev162 | mdg1 | AAGGCGTACATSCTGAATTCGCATATTTAG |
| TEmdg1 Reaction A Nest Rev128 | mdg1 | **GTTCAGACGTGTGCTCTTCCGATCT**AGGGTGTATCTAAAGATCTACTAGGGTGAC |
| TEmdg1 Reaction B For7267 | mdg1 | CTCSACTCCCATTGGTTATCGAGTATG |
| TEmdg1 Reaction B Nest PCR For7427 | mdg1 | **GTTCAGACGTGTGCTCTTCCGATCT**AACCAAATAAAGATAAAATGACCRACTGCG |
| TEmdg3 Reaction A Rev171 | mdg3 | TAGCTTGGTTCAACAGCGACTG |
| TEmdg3 Reaction A Nest PCR Rev108 | mdg3 | **GTTCAGACGTGTGCTCTTCCGATCT**YAGTCGATAAGTTTCTCGATAATATTTGTATG |
| TEmdg3 Reaction B For5302 | mdg3 | ACCCTCTTCCTTTACTCTTAGTCATACATAC |
| TEmdg3 Reaction B Nest PCR For5376 | mdg3 | **GTTCAGACGTGTGCTCTTCCGATCT**ATACACCCATCCTTAACATACAAATATTATCG |
| TEroo Reaction A Rev152 | roo | GATCTCAAGTGACTGACTCATGTAGTGTG |
| TEroo Reaction A Nest Rev116 | roo | **GTTCAGACGTGTGCTCTTCCGATCT**ATTACATGTTTTTGAGCAATGCACCCATG |
| TEroo Reaction B For8964 | roo | CGATRAGGCGGGGACTATTTACKTAGG |
| TEroo Reaction B Nest For8992 | roo | **GTTCAGACGTGTGCTCTTCCGATCT**CTCTGCGTAGGCCATTTACTYTAAGATG |
| TEcopia Reaction A Rev197 | copia | ATTTTAAGTTATTTCAACTGCAACACCAGC |
| TEcopia Reaction A Nest Rev133 | copia | **GTTCAGACGTGTGCTCTTCCGATCT**AGGAAAGAAGGAATAAAAAGAGTGGTATTC |
| TEcopia Reaction B For4863 | copia | TATTATTGTTATGTTTTTAATTATAGACGTTATTTTTGAG |
| TEcopia Reaction B Nest For4967 | copia | **GTTCAGACGTGTGCTCTTCCGATCT**GCCACACCTTTTATGCCATAAAACATATTG |
| I5 | NA | AATGATACGGCGACCACCGAGATC |

**Table 1**: Sequences of the primers used in this study

Nucleotides in bold indicate the Read2 anchor sequence added to the Nest PCRs (Figure 1).
