# Supplementary material for: A novel transposable element-based authentication protocol for Drosophila cell lines
Source: G3 (Bethesda). 2021 Nov 25;12(2):jkab403. doi: 10.1093/g3journal/jkab403 (PMC9210319; doi:10.1093/g3journal/jkab403)
Supplement: jkab403_Supplementary_Data [file jkab403_supplementary_data.zip › GENETICS-G3-2021-402803-s06.docx]

**Supplementary File 1**

**Nested PCR Protocol:**

Step 1:

- Nextera libraries are made by following standard protocol.
- Each library is diluted to 1nM.

Step 2: Reaction A/B (Two sets of reactions)

Reaction A: Primers: i5 + TE Reaction A Rev (To amplify the 5’ flanking region of TE gene)

Reaction B: Primers: i5 + TE Reaction B For (To amplify the 3’ flanking region of TE gene)

2.1 PCR reagents:

| 5X Phusion buffer | 10 μl |
| --- | --- |
| 100 mM dNTP mix | 0.5 μl |
| 100 uM i5 Primer | 0.5 μl |
| 100 uM Reaction A/B (Rev/For) | 0.5 μl |
| Phusion polymerase | 0.5 μl |
| 1nM Library | 5.0 μl |
| ddH_2_O | 33 μl |
| Total | 50 μl |

2.2 PCR settings:

| 98˚C | 30 sec |  |
| --- | --- | --- |
| 98˚C | 10 sec |  |
| 65˚C | 30 sec | 10 cycles |
| 72˚C | 60 sec |  |
| 72˚C | 5 min |  |
| 4˚C | Hold |  |

2.3 Cleaned with 0.9X AMPure XP beads, washed with 80% ethanol twice, and elute with 40 μl Elution Buffer (EB).

**Step 3:** Nest PCR (Two sets of reactions)

Set 1: Primers: i5 + TE Reaction A Nest PCR Reverse (Template: Reaction A products)

Set 2: Primers: i5 + TE Reaction B Nest PCR Forward (Template: Reaction B products)

3.1 PCR reagents:

| 5X Phusion buffer | 10 μl |
| --- | --- |
| 100 mM dNTP mix | 0.5 μl |
| 100 uM i5 Primer | 0.5 μl |
| 100 uM NestPCR Reaction A/B (Rev/For) | 0.5 μl |
| Phusion polymerase | 0.5 μl |
| Reaction A/B products | 38 μl |
| Total | 50 μl |

3.2 PCR setting:

| 98˚C | 30 sec |  |
| --- | --- | --- |
| 98˚C | 10 sec |  |
| 65˚C | 30 sec | 10 cycles |
| 72˚C | 60 sec |  |
| 72˚C | 5 min |  |
| 4˚C | Hold |  |

3.3 Cleaned with 0.9X AMPure XP beads, wash with 80% ethanol twice, and eluted with 19 μl EB.

**Step 4:** Index adding PCR with NEBNext 6609 Primers

4.1 PCR reagents:

| 5X Phusion buffer | 10 μl |
| --- | --- |
| 100 mM dNTP mix | 0.5 μl |
| 100 uM i5 Primer | 0.5 μl |
| NEBNext 6609S Primer | 5 μl |
| Phusion polymerase | 0.5 μl |
| Nest PCR Reaction A + B products | 33.5 μl |
| Total | 50 μl |

4.2 PCR settings:

| 98˚C | 30 sec |  |
| --- | --- | --- |
| 98˚C | 10 sec |  |
| 65˚C | 30 sec | 3 cycles |
| 72˚C | 60 sec |  |
| 72˚C | 5 min |  |
| 4˚C | Hold |  |

4.3 Cleaned with 0.8X AMPure XP beads, washed with 80% ethanol twice, and eluted with 32 μl of EB.
