# Supplementary material for: A novel transposable element-based authentication protocol for Drosophila cell lines
Source: G3 (Bethesda). 2021 Nov 25;12(2):jkab403. doi: 10.1093/g3journal/jkab403 (PMC9210319; doi:10.1093/g3journal/jkab403)
Supplement: jkab403_Supplementary_Data [file jkab403_supplementary_data.zip › GENETICS-G3-2021-402803-s07.docx]

**Supplementary methods**

*Sample Processing and Transposable Element Identification – CB Lab, UGA pipeline*

1. *Merge non-multiplexed data into multiplexed data*

The TE-NGS pipeline treats the products of TE-NGS sequencing run as multiplexed Illumina paired-end reads (R1 = read 1, R2 = read 2) including data from Nest PCR Reaction A and Reaction B for all six focal TE families. In the event that the input data type is Nest PCR Reaction A and B, non-multiplexed or TE family non-multiplexed Illumina paired-end data with multiple fastq.gz files for R1 and R2, then the analysis pipeline would merge input data into a single R1 and R2 before subsequent processing.

1. *Align R1 and R2 to modified* D. melanogaster *reference genome*

TE family and reaction multiplexed R1 and R2 fastq files were next mapped to a “masked augmented” version of the *D. melanogaster* reference genome, which was generated as follows:

a) Downloaded the unmodified dm6 from UCSC (<http://hgdownload.soe.ucsc.edu/goldenPath/dm6/bigZips/dm6.fa.gz>).

b) Extracted and retained chr2L, chr2R, chr3L, chr3R, chr4, chrM, chrY and chrX from unmodified dm6

c) Hard masked TE sequences in dm6 reference genome using repeatmasker v4.0.7 (<https://github.com/bergmanlab/transposons/blob/master/current/D_mel_transposon_sequence_set.fa>).

d) Added canonical TE sequences as contigs to the end of masked dm6 fasta sequence.

These modifications of the reference genome resulted in all reads matching TE sequences to map to canonical TE sequence contigs rather than TE sequences distributed throughout the refence genome. Sorted, indexed R1 and R2 BAM file were generated as outputs from this step.

1. *Demultiplex mapped reads by TE family and by Nest PCR reaction*

The analysis pipeline then demultiplexed R1 reads mapped to unique regions of the genome by scanning R2 reads from the same sequenced fragment that map to canonical TE sequences. Each read pair from a fragment was assigned to a specific TE family and Nest PCR reaction (A/B) based on the TE family and strand information in the R2 mapping relative to the canonical TE sequence. TE family demultiplexing for R1 mapped reads is based on the canonical TE sequence matched in the corresponding R2 alignment from the same fragment. NestPCR2 reaction demultiplexing for R1 mapped reads was based on the strand to which the respective R2 read matches, relative to the canonical TE sequence (Figure S1 A). If R2 read was mapped to the forward strand of the canonical TE sequence, then both R2 and the paired R1 were assigned as Nest PCR reaction B; If R2 read was mapped to the reverse strand of the canonical TE sequence, then both R2 and the paired R1 were assigned to Nest PCR reaction A. Multiple R1 BAM files were generated from the workflow at this step, including the TE-specific and Nest Reaction mapping data for each R1 (Figure S1B).

1. *Identify TE insertions based on demultiplexed mapped R1 reads.*

For each TE family, demultiplexed Reaction A and Reaction B R1 BAM files were then processed pair-wise to detect TE insertions (Figure. S1C). The TE detection workflow consists of following steps:

a) Per-base depth profile from the Reaction A and Reaction B R1 BAM files was generated separately.

b) For each depth profile, positions with low depth of coverage were filtered out using a TE family and Nest PCR reaction specific depth cutoff threshold.

c) For each filtered depth profile, the remaining positions were merged into TE enriched cluster annotation using bedtools v2.29.2 (window: 100bp).

d) Reference TEs were identified by searching for TE cluster annotations from Reaction A and Reaction B that are close to a reference TE annotation on dm6 (window: 100bp). The reference TE annotation used was downloaded and converted from flybase (release r6.29). A reference TE will be reported if TE clusters can be found on both side of the curated reference TE annotation (Figure S1D).

e) Non-reference TEs were identified by intersecting TE cluster annotations from Reactions A/B and searching for overlaps between clusters in the two sets. A non-reference TE was reported if the overlap length between Reaction A/B annotations was between 0-12bp (typical length of target site duplication created by LTR retrotransposon insertions, Figure S1E).

f) Finally, non-reference TEs were filtered by the pipeline to exclude predictions in low recombination regions using defined boundaries (Cridland *et al.* 2013). This step eliminates regions that are dense in repetitive DNA that may cause false positive predictions.

1. *Cluster samples based on non-reference TE predictions*

Non-reference TE predictions in normal recombination regions from multiple samples were then sorted and clustered using bedtools v2.29.2 (window: 10bp, strand independent). Positionally clustered non-reference TE predictions were then converted to a binary presence/absence matrix and a neighbor joining dendrogram is constructed from the binary matrix (distance measure: euclidean) to cluster samples by similarity in TE insertion content.
