# Supplementary material for: A novel transposable element-based authentication protocol for Drosophila cell lines
Source: G3 (Bethesda). 2021 Nov 25;12(2):jkab403. doi: 10.1093/g3journal/jkab403 (PMC9210319; doi:10.1093/g3journal/jkab403)
Supplement: jkab403_Supplementary_Data [file jkab403_supplementary_data.zip › GENETICS-G3-2021-402803-s09.docx]

***Supplementary Legends***

***Supplementary Figure 1***: **Clustering of cell lines based on genomic transposable element distribution using an alternative bioinformatics pipeline.** The cell line clustering is derived from processing NGS data as described in Supplementary File **2**. The triplicates for each cell lines are indicated with 1-3 following the cell line name.

***Supplementary Figure 2***: **Unique TEs distinguish cell lines assessed by gTED.** The number of TEs that are shared between the samples (Intersection size) are plotted in this UpSET plot. Filled in dots indicate the samples that share the particular set of TEs. The absolute number of TEs for each of the samples is plotted as Set Size.

***Supplementary Figure 3***: **Blinded samples have unique gTED signatures.** External (**A**) and internal (**B**) blinded samples assessed using the gTED protocol have unique gTED signatures that cluster replicates by cell identity.

***Supplementary Figure 4***: **S2R+ cells retain unique gTED signature despite extensive passaging.** All samples from this study assessed using the gTED protocol indicate that all the S2R+ passages cluster together, still retaining a unique cell-line specific gTED signature. The S2R+ passages are shaded in green.

***Supplementary File 1: Description of the Nested PCR protocol***

***Supplementary File 2***: **Description of** **the alternative** **bioinformatics** **pipeline used to cluster cell lines based on genomic transposable element distribution.** Clustering using this alternative approach for cell lines used in the development phase of the project is shown in Supplementary Figure 1.

***Supplementary File 3***: **Table of samples ID listed in SRA accession used for gTED analysis.** The 75 samples used for the analysis in the manuscript are listed in the table. The other 39 samples listed in SRP323476 were used for testing and development.

***Supplementary File 4***: **Presence absence matrix for cell line clustering.** The final data matrix used for cell line clustering is available at: <https://github.com/mondegreen/DrosCellID/blob/main/combined.presence-absence.example.tsv>.
