# Supplementary figures and images for: A novel transposable element-based authentication protocol for Drosophila cell lines
Source: G3 (Bethesda). 2021 Nov 25;12(2):jkab403. doi: 10.1093/g3journal/jkab403 (PMC9210319; doi:10.1093/g3journal/jkab403)

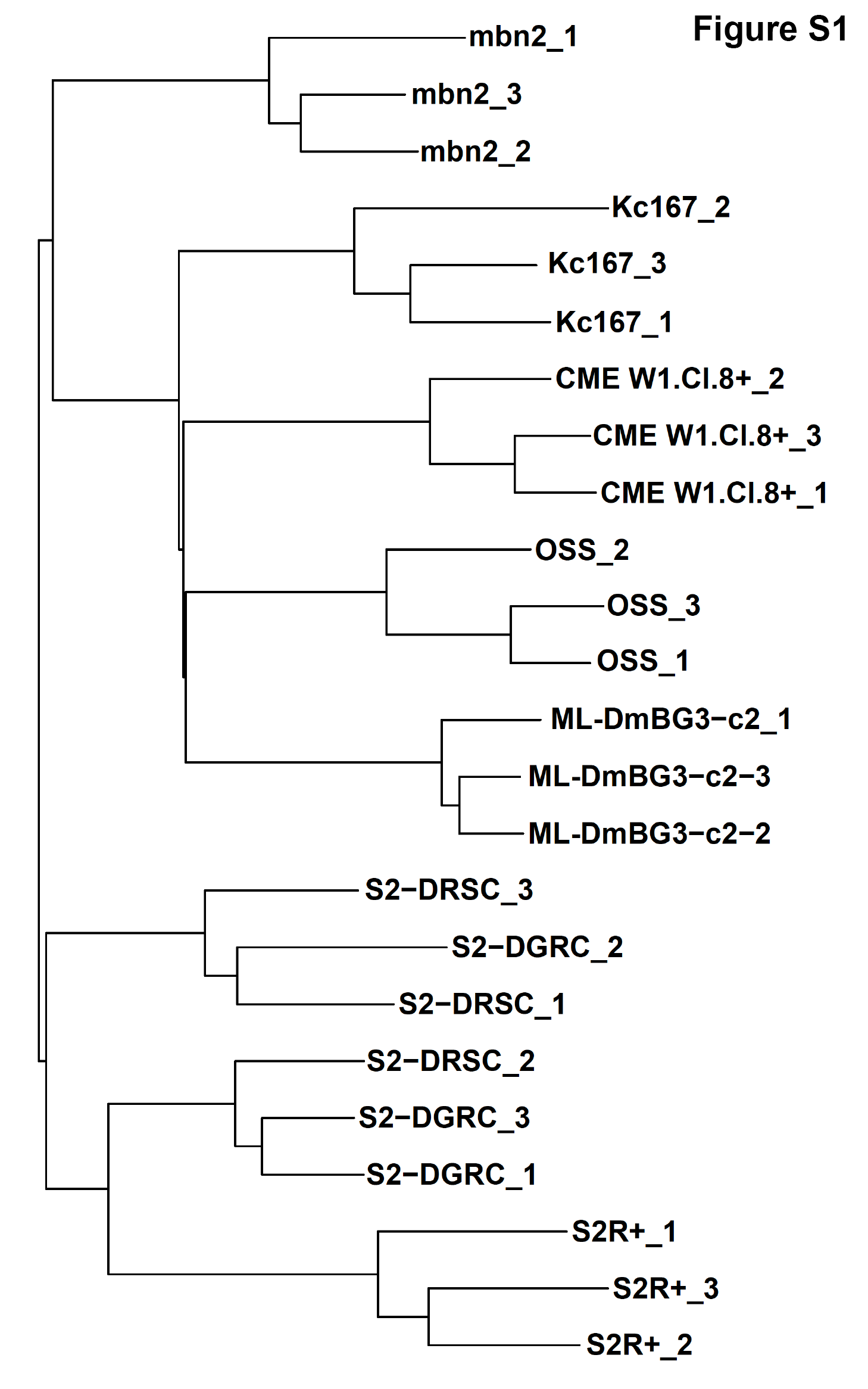

Supplement: jkab403_Supplementary_Data [file jkab403_supplementary_data.zip › GENETICS-G3-2021-402803-s01.tif]

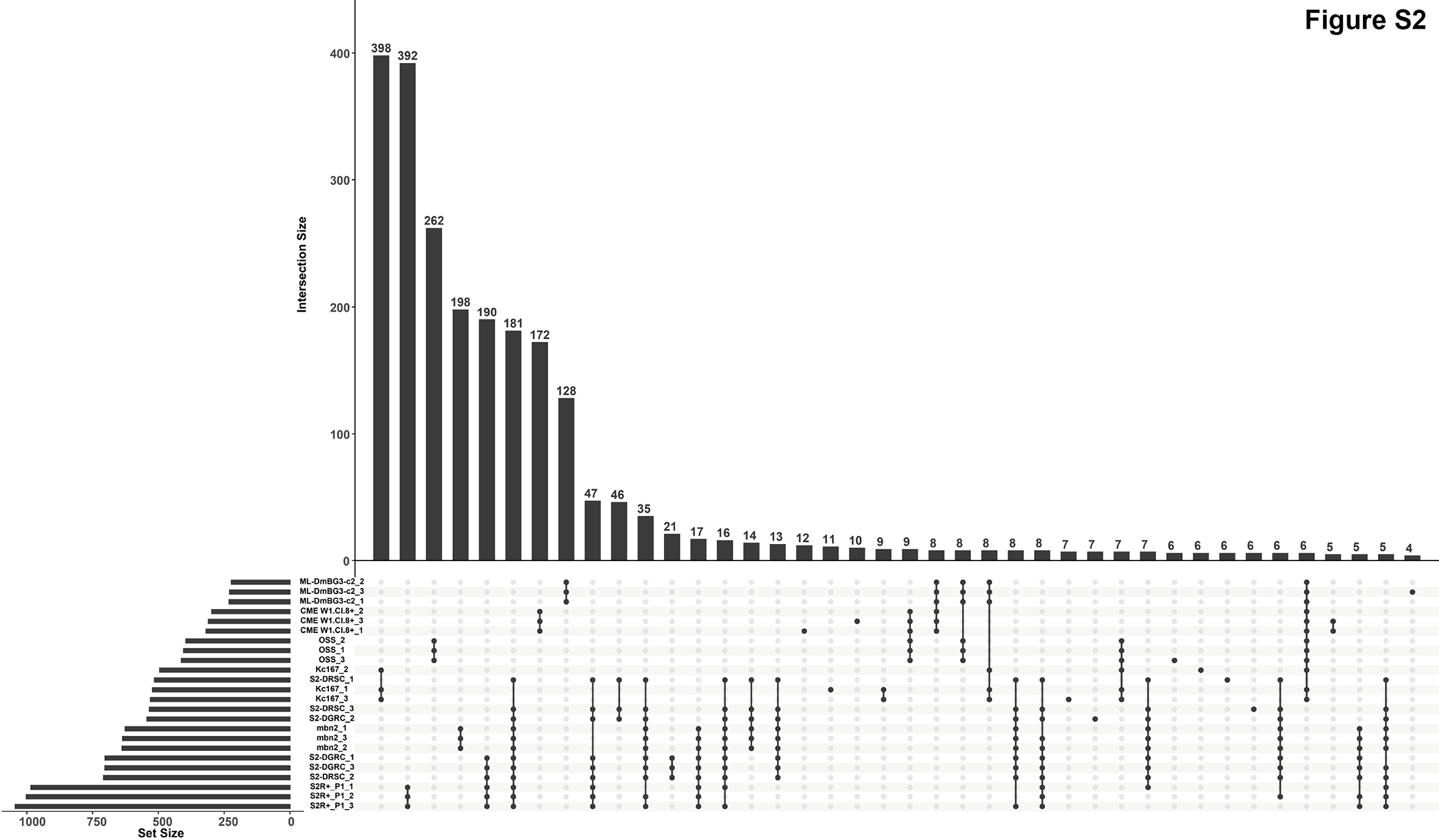

Supplement: jkab403_Supplementary_Data [file jkab403_supplementary_data.zip › GENETICS-G3-2021-402803-s02.tif]

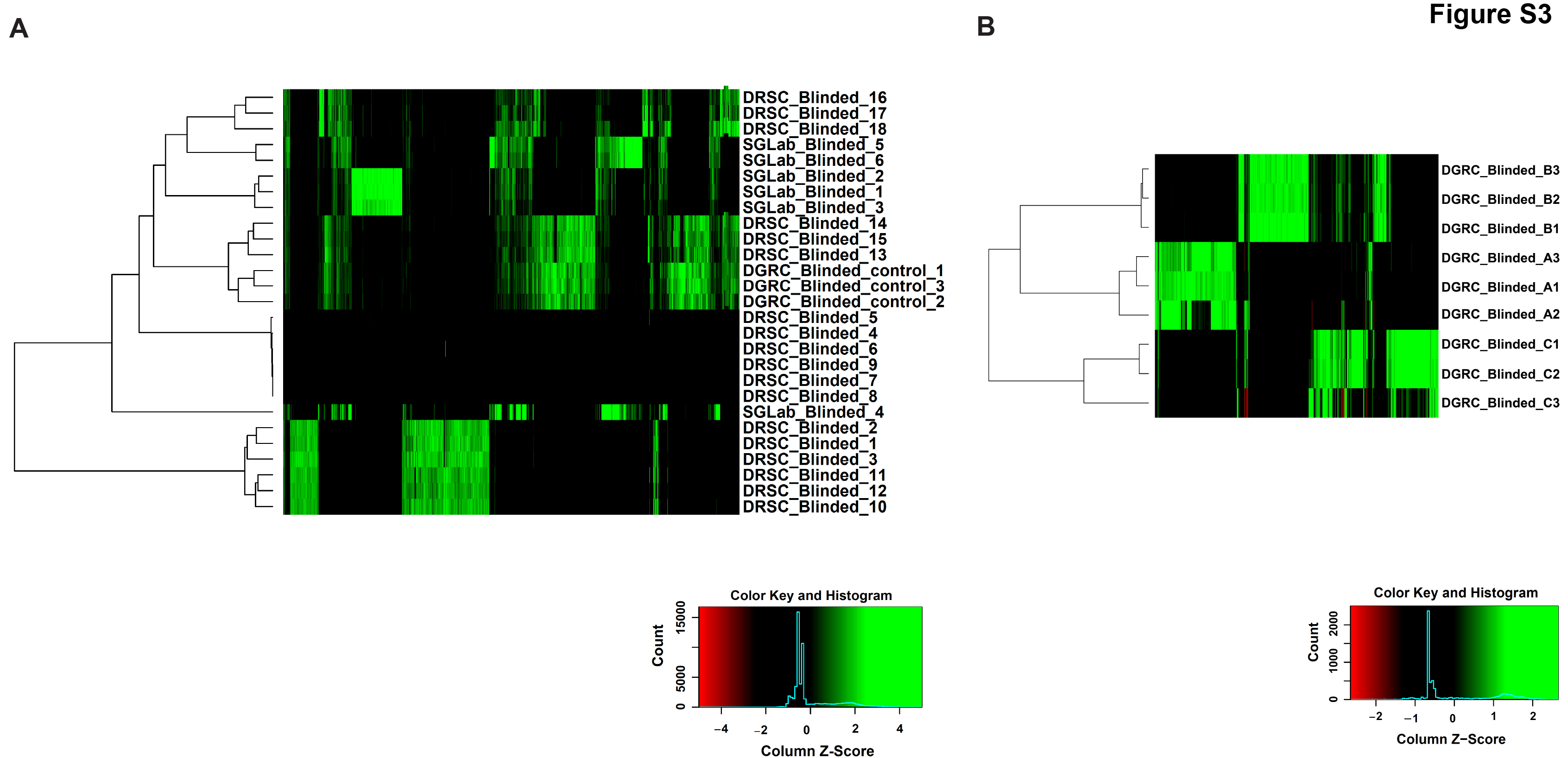

Supplement: jkab403_Supplementary_Data [file jkab403_supplementary_data.zip › GENETICS-G3-2021-402803-s03.tif]

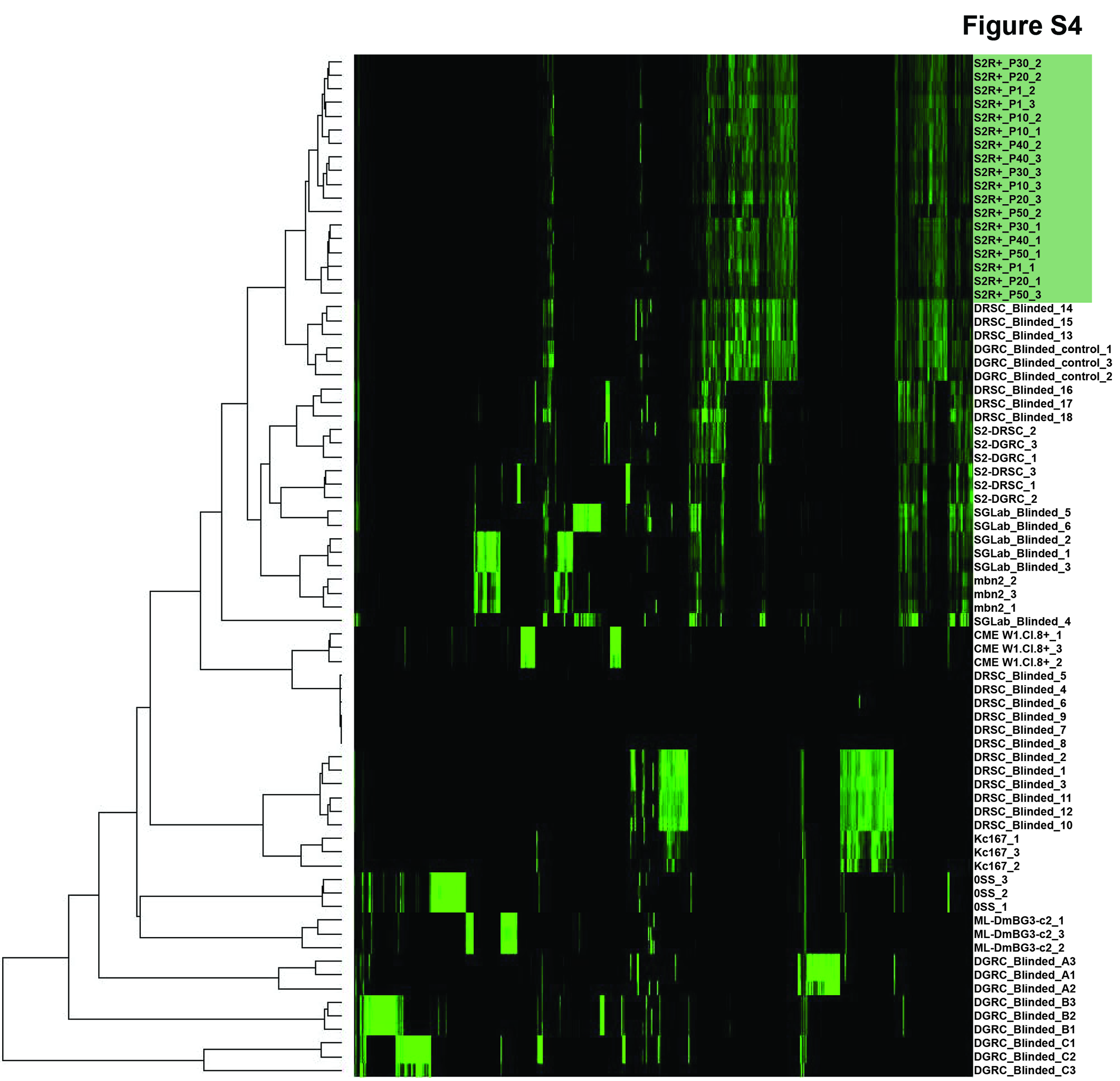

Supplement: jkab403_Supplementary_Data [file jkab403_supplementary_data.zip › GENETICS-G3-2021-402803-s04.tif]
